# Mediterranean Plants with Antimicrobial Activity against *Staphylococcus aureus*, a Meta-Analysis for Green Veterinary Pharmacology Applications

**DOI:** 10.3390/microorganisms11092264

**Published:** 2023-09-09

**Authors:** Francesca Oppedisano, Rosario De Fazio, Enrico Gugliandolo, Rosalia Crupi, Ernesto Palma, Sayed Haidar Abbas Raza, Bruno Tilocca, Carmine Merola, Cristian Piras, Domenico Britti

**Affiliations:** 1 Institute of Research for Food Safety & Health (IRC-FSH), Department of Health Sciences, “Magna Græcia University” of Catanzaro, Campus Universitario “Salvatore Venuta” Viale Europa, 88100 Catanzaro, Italy; foppedisano@unicz.it (F.O.); palma@unicz.it (E.P.); 2 Department of Health Sciences, “Magna Græcia University” of Catanzaro, Campus Universitario “Salvatore Venuta” Viale Europa, 88100 Catanzaro, Italy; rosario.defazio@studenti.unicz.it (R.D.F.); tilocca@unicz.it (B.T.); britti@unicz.it (D.B.); 3 Department of Veterinary Science, University of Messina, 98166 Messina, Italy; egugliandolo@unime.it (E.G.); rcrupi@unime.it (R.C.); 4 Guangdong Provincial Key Laboratory of Food Quality and Safety, Nation-Local Joint Engineering Research Center for Machining and Safety of Livestock and Poultry Products, South China Agricultural University, Guangzhou 510642, China; haiderraza110@scau.edu.cn; 5 Department of Bioscience and Technology for Food Agriculture and Environment, University of Teramo, Via Balzarini 1, 64100 Teramo, Italy; cmerola@unite.it; 6 CISVetSUA, University of Catanzaro, Campus Universitario “Salvatore Venuta” Viale Europa, 88100 Catanzaro, Italy

**Keywords:** Mediterranean plants, antimicrobial plant extracts, essential oil, *Staphylococcus aureus*

## Abstract

Antimicrobial resistance (AMR) has emerged as a global health crisis, necessitating the search for innovative strategies to combat infectious diseases. The unique biodiversity of Italian flora offers a treasure trove of plant species and their associated phytochemicals, which hold immense potential as a solution to address AMR. By investigating the antimicrobial properties of Italian flora and their phytochemical constituents, this study aims to shed light on the potential of phyto-complexes as a valuable resource for developing novel or supportive antimicrobial agents useful for animal production.

## 1. Introduction

Antimicrobial resistance has become a pressing global challenge, rendering conventional antibiotics less effective and diminishing our ability to combat infectious diseases. The urgency to counter this crisis has led to a growing interest in alternative sources, including plant-derived compounds. Italian flora, with its wealth of botanical species, represents an untapped source of diverse phytochemicals that may possess potent antimicrobial properties [1].

The Italian flora exhibits remarkable heterogeneity due to the country’s diverse geography, climate, and historical influences. Italy is home to a wide range of ecosystems, including the Alpine region in the north, the Apennine Mountains that run through the center of the country, coastal areas, wetlands, and Mediterranean islands. This diverse landscape contributes to the rich and varied plant life found throughout Italy [2].

The central regions are dominated by the Apennine Mountains, which harbor a unique array of flora. The Apennines support a mix of deciduous and evergreen forests, including oak, beech, chestnut, and pine. The Mediterranean influence becomes more pronounced in the central and southern parts of the country, with characteristic maquis shrubland, macchia, and coastal vegetation. Plants such as rosemary (*Rosmarinus officinalis*), lavender (*Lavandula* spp.), myrtle (*Myrtus communis*), and various aromatic herbs thrive in these Mediterranean ecosystems [3].

Coastal regions of Italy boast their own distinct flora, shaped by the proximity to the sea and the specific microclimates along the shoreline. Coastal dunes and wetlands are home to salt-tolerant plants, such as sea lavender (*Limonium* spp.), sea holly (*Eryngium* spp.), and various species of halophytes adapted to saline conditions. The wetlands of the Po Delta, Venice Lagoon, and other marshy areas support a diverse range of aquatic and semi-aquatic plants, including reeds, cattails, and water lilies [4].

The islands of Sicily and Sardinia, located in the Mediterranean Sea, have their own unique flora due to their isolation and climatic conditions. These islands exhibit a blend of Mediterranean and North African influences, resulting in a diverse mix of plant species. Iconic plants like the Sicilian fir (*Abies nebrodensis*), dwarf palm (*Chamaerops humilis*), and Sardinian broom (*Genista corsica*) can be found in these regions [5].

Phyto-complexes derived from Italian flora offer a complex mixture of bioactive compounds, including alkaloids, flavonoids, terpenes, and phenolics. These phytochemicals have evolved as part of plants’ defense mechanisms against microbial infections and environmental stresses, providing a wide array of chemical structures and mechanisms of action. By harnessing the antimicrobial potential of these phyto-complexes, it may be possible to address the challenge of antimicrobial resistance through novel therapeutic interventions.

Such investigations have revealed promising outcomes, demonstrating the ability of phytochemicals derived from Italian plants to inhibit the growth of various pathogenic microorganisms, including multidrug-resistant strains. These bioactive compounds can target essential microbial processes, disrupt cellular structures, and interfere with virulence factors, thus reducing the risk of resistance development [1].

Various intervention methods can be employed to prevent the spread and dangers associated with antimicrobial resistance. Certainly, the first valuable approach is tied to the research for early diagnosis through several methods, such as immunoproteomics or high throughput mass spectrometry techniques [6,7,8,9]. Other approaches involve isolating and removing animals exhibiting recurring resistance patterns in their microbiomes. Alternatively, a strategy involves implementing Green Veterinary Pharmacology (GVP) practices [1,10,11], utilizing crops and plants that produce antibacterial molecules. The Italian territory, with its abundant biodiversity of native plants boasting diverse nutraceutical functions, offers a promising resource for this purpose [12]. Some of this knowledge is rooted in the ancient traditions of rural areas and could be reassessed through scientific methods to validate any potential antimicrobial activity [13]. Another portion of this knowledge is already documented in scientific literature and requires systematic review. Assessing the efficacy of these plants or their extracts against microbes that jeopardize animal production efficiency could serve as a viable alternative to conventional antimicrobial therapeutic approaches. Furthermore, it may aid in curbing the emergence of additional antibiotic resistance phenomena.

This manuscript aims to delve into the vast repertoire of Italian flora and its potential as a solution to antimicrobial resistance. By reviewing the existing literature on the antimicrobial properties of plant species and their phytochemical constituents, we seek to identify key plant species and compounds that show the highest MIC values against *S. aureus*. This was performed via a systematic literature review and a meta-analysis of the MIC values recorded in the analyzed literature.

## 2. Materials and Methods

The bibliographic searches were performed following the list of plants detected by Piras et al. [1]. The work described by the authors provided a ready-to-use comprehensive list of plants to be further tested against the indicated list of pathogens and to provide new alternative strategies against bacterial pathogens to be employed in Green Veterinary Pharmacology applications.

The list of plant extracts active against the most relevant pathogens for animal husbandry and animal infections was published in 2022 [1]. The list of plants growing in Italian territory was organized in a pathogen-driven way and included Gram+ and Gram- bacteria. Moreover, 39 plants were reported to be active against *S. aureus*, posing the query about which ones have the lowest MICs. Herein, in order to understand which ones may be suitable for a GVP approach, a new systematic review approach was used to register the MIC values so far annotated in the literature. The new searches were performed with no time nor geographic restriction in order to gather the most complete dataset.

From methodological point of view, all the detected and annotated plants as in the following Table 1 (adapted from Piras et al. [1]) and recorded as active against *S. aureus* were searched, using their scientific name and “*Staphylococcus aureus*” (e.g., *Cinnamomum camphora* and *Staphylococcus aureus*), again in PubMed, web of science and Scopus. All the entries were saved in separate files for each search. All the files (three for each plant) were uploaded on Rayyan (https://www.rayyan.ai/, accessed on 19 January 2023) and afterward exported as a single file in the .ris format. This step was performed in order to merge all the search results in a single file. The obtained file was afterward uploaded on Mendeley desktop (version 1.19.8) for the manual check and the duplicates removal. The obtained filtered file was exported and afterward uploaded in Rayyan (https://www.rayyan.ai/, accessed on 19 January 2023) for the keywords search and for the screening of the relevant manuscripts to be enrolled in the meta-analysis.

The keywords for the research on Rayyan were “MIC”, “MICs”, and “plant scientific name” (*Genre species).* This method allowed, for each plant, the detection of the scientific manuscripts where the words “MIC” and “MICs” were mentioned in the title/keywords/abstract, and, presumably, the MIC was studied/evaluated. After this selection process, every record was manually evaluated for MIC detection. In case the MIC value against *S. aureus* was clearly indicated, the study was included. This process was done individually by three different independent reviewers; the results were validated by cross-validating 15 randomly chosen recorded values by another referee. The MIC values for each plant, recorded in each independent study, were annotated in an Excel file along with the manuscript title. The mean and SD were calculated within the same spreadsheet, and obtained results were further analyzed with OpenMeta[Analyst] (http://www.cebm.brown.edu/openmeta/, accessed on 19 January 2023) for the creation of the Forest plots.

## 3. Results

**Figure 1:**
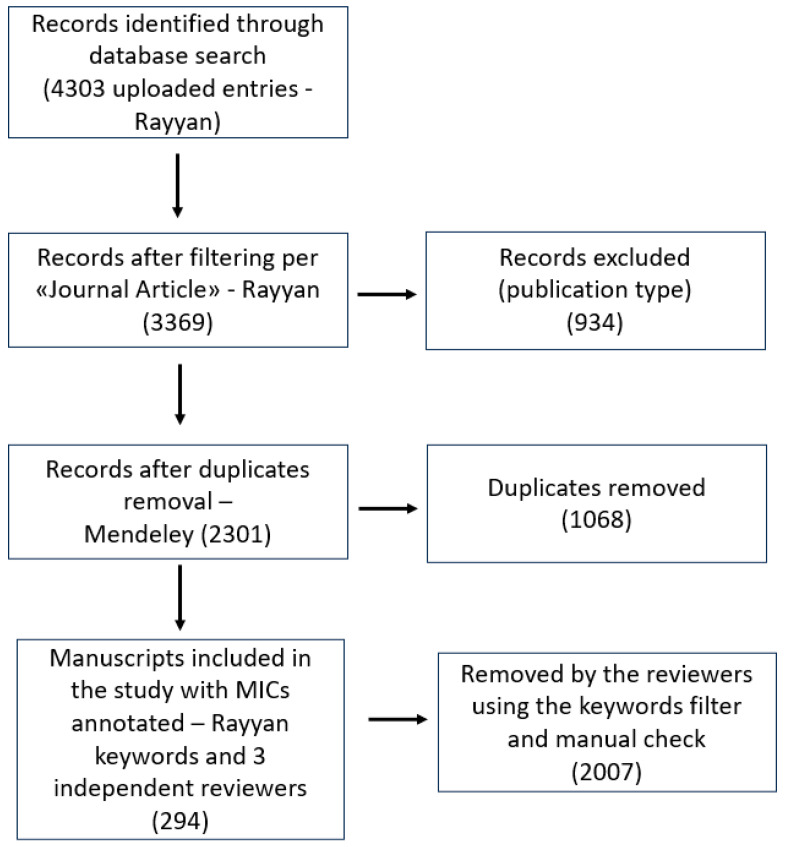
Prisma diagram illustrating the records and the filtering steps performed before the manual reviewing process of the abstracts.

All the plants annotated in the previously published review were individually searched against *S. aureus* in three different databases (see methods section). The search produced 4303 entries; reviews and other article types were excluded using Ryyan, resulting in 3369 entries. All other duplicates were removed, with Mendeley yielding 2301 records whose abstracts were manually screened by three independent reviewers for the MICs detection. The workflow regarding records inclusion and filtering is shown in the following Figure 1.

The records for each plant were further screened with the keywords for the inclusion and exclusion system of Rayyan. The keywords used for the records filtering were “MIC”, “MICs”, and “plant name”. All the resulting records were then manually screened for the MIC annotation. The results of this filtering process done for each plant are presented in Table 2. The list of annotated MICs, along with the DOI and the title of the manuscripts is visible in the Table S1 (Excel file, Supplementary Materials).

**Figure 2:**
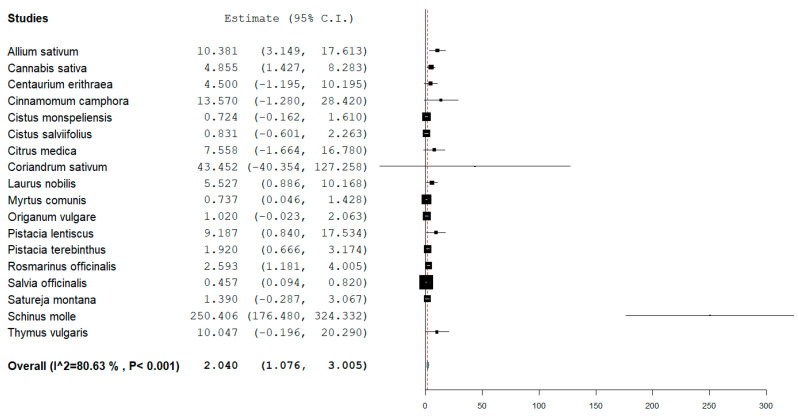
Forest plot showing the representation of the average recorded MICs for each plant extract included in this study. The power (that is related to the number of studies considered) is indicated by the weight (size) of the box.

The quantitative MICs evaluation produced the results shown in Figure 2. Extracts of plants such as *Coriandrum sativum, Crocus sativus*, and *Schinus molle* showed high MIC values up to mean values as high as 200 mg/mL.

**Figure 3:**
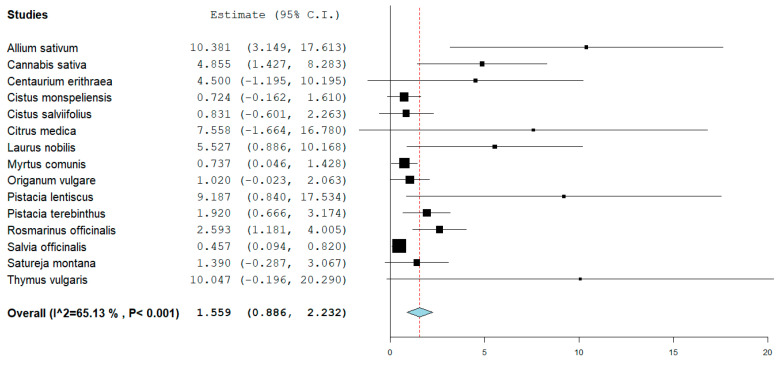
Forest plot showing the representation of the average recorded MICs for the most effective plant extracts included in this study. For the benefit of visualization purposes, the plants with the highest MICs were excluded from this plot. The power (that is related to the number of studies considered) is indicated by the weight (size) of the box.

For a better visualization, the plants reporting the highest MICs were removed from the forest plot and, as visible in Figure 3, the most effective plant extracts were *Salvia officinalis*, *Cistus monspeliensis*, *Cistus salviifolius*, *Origanum vulgare,* and *Myrtus communis* with recorded average MICs against *S.aures* of, respectively, 0.46 (±0.56) mg/mL, 0.72 (±0.78) mg/mL, 0.83 (±1.03) mg/mL, 1.02 (±3.28), and 0.74 (±1.32).

## 4. Discussion

*S. aureus* is a significant human pathogen as well as a pathogen that is the causative agent of animal illnesses/conditions [48]. This bacterium causes mastitis in dairy cattle, an udder infection that causes major economic losses worldwide [49]. Antibiotic therapy is routinely used by veterinarians to treat infections, which increases the possibility of antibiotic resistance over time [50]. Intramammary dosing of a penicillin–novobiocin combination used to treat mastitis has been linked to increasing antimicrobial resistance, especially to ampicillin [51]. Other investigations conducted in different countries found that resistance to ampicillin ranged between 5.2% and 77.3% for bovine *S. aureus* strains, between 0% and 44.2% for gentamicin, and between 3% and 60% for tetracycline. In this regard, novel strategies to minimize resistance development are being developed [52].

The Mediterranean region is renowned for its abundant plant biodiversity, which has been cultivated and utilized for centuries in traditional medicine. Among the numerous therapeutic properties attributed to Mediterranean plants, their antimicrobial activity stands out as an important attribute [53]. The unique ecological conditions, including a mild climate and diverse soil composition, have contributed to the rich diversity of phytochemicals in these plants, which possess remarkable potential in combating microbial pathogens [1]. This serves as a tool to fight antimicrobial resistance, which has become a critical global health challenge [48]. The complex chemical composition of these plants, including alkaloids, terpenes, flavonoids, phenolics, and essential oils, provides a diverse range of compounds with diverse mechanisms of action against a broad spectrum of microbial pathogens, including bacteria, fungi, and viruses [54,55].

The antimicrobial mechanisms employed by these phytochemicals encompass various actions, such as disruption of cell membranes, inhibition of enzyme activity, modulation of microbial gene expression, and interference with microbial adhesion and biofilm formation [56,57,58].

Numerous plants have been investigated for their bioactive compounds and their potential efficacy against *Staphylococcus aureus*, including both methicillin-susceptible *Staphylococcus aureus* (MSSA) and methicillin-resistant *Staphylococcus aureus* (MRSA) strains [1,59]. It is important to note that the effectiveness of these plants and their extracts can vary depending on the specific strains of *Staphylococcus aureus* tested, the concentration and formulation of the extracts, and the methods used for evaluation. For this reason, the literature on this subject is very heterogeneous, and it is missing a work capable of summarizing the plant extracts showing the highest MICs against the growth of *S. aureus* [56].

This research holds significant relevance, primarily because MRSA is the central concern within the antimicrobial resistance (AMR) monitoring initiative for animals raised for food production. Consequently, the proliferation of these bacteria resistant to multiple drugs presents a considerable public health hazard, given the potential for cross-species transmission, including to humans [60].

As described in the results section, we performed a systematic literature search and filtering that produced 2301 records that were subsequently manually filtered (see methods section) and screened by three different reviewers. The second column of Table 2 shows the total records for each plant, and the third column shows the records whose MIC was annotated for further statistical analysis. The forest plot represented in Figure 2 shows all the plants involved in this study, including plants such as *Schinus molle*, *Coriadrum sativum,* and *Crocus sativus,* which showed high values of average MICs. It is important to note that while *Schinus molle* has demonstrated antibacterial activity in various studies, further research is still needed to determine the optimal extraction methods, identify the most potent bioactive compounds, and evaluate the plant’s potential for clinical applications, but for the purpose of this work, it was discarded from further analysis. Additionally, the concentration, formulation, and mode of application of *Schinus molle* extracts can significantly influence their antibacterial efficacy. The average MIC calculated from the MICs recorded in our included studies was equal to ≈250 mg/mL, and the recorded values ranged from 176 to 324 mg/mL; therefore, we can consider that those values are too high to be considered a potential candidate against *S. aureus*. Even with lower MICs, plants such as *Coriandrum sativum* and *Crocus sativus* showed high average values (≈43 mg/mL and ≈24 mg/mL, respectively). For a more comprehensive visualization, those plants were removed from the forest plot.

The forest plot shown in Figure 3 does not include the three previously mentioned plant extracts and shows the most effective ones. 

*Salvia officinalis* [52,61,62,63,64,65,66,67,68], *Cistus monspeliensis* [69,70,71,72], *Cistus salviifolius* [69,71], *Origanum vulgare* [70,73,74,75,76,77,78,79,80,81,82,83,84,85,86,87,88,89,90,91,92,93,94,95,96,97,98,99,100,101,102,103,104,105,106,107], and *Myrtus communis* [19,108,109,110,111,112,113,114,115,116,117,118,119,120] showed an average MIC against *S. aureus* of respectively 0.46 (±0.56) mg/mL, 0.72 (±0.78) mg/mL, 0.831 (±1.033) mg/mL, 0.997 (±3.24), and 0.736 (±1.32).

*Myrtus communis*, commonly known as common myrtle or true myrtle, is a flowering plant native to the Mediterranean region. It has been used for various medicinal purposes throughout history, and its essential oil was used as an antibacterial [121,122]. It possesses antibacterial activity against Gram-positive and Gram-negative and is particularly active against *Escherichia coli*, *Staphylococcus aureus*, *Salmonella typhimurium*, and *Pseudomonas aeruginosa* [123]. Monoterpenes are the most abundant group of compounds in *Myrtus communis* essential oil. They include compounds such as α-pinene, β-pinene, limonene, myrcene, and cineole (also known as eucalyptol) [124,125]. These compounds contribute to the characteristic scent of the essential oil and have shown various biological activities, including antibacterial and anti-inflammatory. Sesquiterpenes are another group of compounds present in *Myrtus communis* essential oil, although in smaller amounts compared to monoterpenes [126]. Examples of sesquiterpenes found in *Myrtus communis* essential oil include caryophyllene, germacrene D, and α-humulene [127]. Sesquiterpenes are known for their potential anti-inflammatory and antioxidant properties. Oxides, such as 1,8-cineole (eucalyptol), are commonly found in *Myrtus communis* essential oil. Oxides can contribute to the expectorant and respiratory benefits of the essential oil and may have antimicrobial properties [112,128].

Sage exhibits antimicrobial activity against both methicillin-resistant *Staphylococcus aureus* (MRSA) and methicillin-susceptible *Staphylococcus aureus* (MSSA) strains [129]. Its extracts contain compounds such as thujone, camphor, and cineole. These compounds have been shown to possess antibacterial properties and can inhibit the growth and proliferation of bacteria [67,130].

*Cistus monspeliensis*, commonly known as Montpellier cistus or rock rose, is a Mediterranean shrub known for its medicinal properties. It has been traditionally used for various therapeutic purposes, including its potential antimicrobial activity against *Staphylococcus aureus* [131]. While there is limited specific research available on the antibacterial activity of *Cistus monspeliensis* against *Staphylococcus aureus*, other species of the Cistus genus have been studied for their antimicrobial properties [132]. The antimicrobial activity of Cistus species is believed to be attributed to their bioactive compounds, such as polyphenols, flavonoids, and tannins, that are effective antibacterial agents [133].

The herb oregano, also known as *Origanum vulgare* L., is a perennial aromatic plant that is a member of the Lamiaceae family. It is frequently used as a flavoring herb, feed addition, and garden adornment, but it also serves as a medicine since it possesses antibacterial, antioxidant, and anti-inflammatory effects [134,135]. Oregano has a wide range of uses in food preservation due to the abundance of its active ingredients, including carvacrol and thymol, but it also has a unique bacteriostatic action on *Salmonella enteritidis* [136], *Cronobacter sakazakii* [137], *Escherichia coli* [135,138,139], *Staphylococcus aureus* [135], and *Listeria monocytogenes* [140,141]. There are differences in the quantity and quality of oregano essential oil (OEO) from diverse germplasm sources. The molecular composition of OEOs from various populations differs noticeably even under the same development environment. The composition of plant essential oils, which affects their antibacterial qualities, is influenced by a number of factors in addition to the diverse plant species, including the geographical environment and the time of harvest. With a rise in demand for the plant, artificial oregano growth and cultivar development have become more significant [137].

The five plants, *Salvia officinalis* (Common Sage), *Cistus monspeliensis* (Montpelier Rockrose), *Cistus salviifolius* (Sage-leaved Rockrose), *Origanum vulgare* (Common Oregano), and *Myrtus communis* (Common Myrtle), belong to different genera but share common traits in their composition. These traits provide valuable insights into their botanical classification and evolutionary relationships. All of these plants belong to the Kingdom Plantae and are classified under the Division Magnoliophyta (angiosperms) since they are flowering plants. In the case of *Salvia officinalis* and *Origanum vulgare,* they belong to the Lamiaceae family (the mint family), and both are part of the Mentheae tribe. *Cistus monspeliensis* and *Cistus salviifolius* are members of the Cistaceae family. There is significant research attention focused on the Cistus L. genus, which comprises numerous plants utilized in traditional medicine by populations residing in the vicinity of the Mediterranean Sea [142,143]. Myrtus communis belongs to the Myrtaceae family. Plants in this family are characterized by their aromatic leaves, usually containing essential oils, and their flowers often have numerous stamens surrounding a central style [144].

Despite being members of different genera, some of these plants share common traits in their taxonomy; two are part of the Lamiaceae family and two are of the Cistaceae family. Only *Myrtus communis* belongs to the Myrtaceae family.

From a chemical composition point of view, according to El Euch and colleagues [145], *S.officinalis* EO is mainly composed of Camphor (33.61%), 1,8-cineole (22.22%), and α-thujone (21.43%); the most abundant compounds detected in *Cistus* spp. include α-pinene, viridiflorol, borneol, trimethyl cyclohexanone, and camphene [146]; *Origanum* spp. essential oil is mainly composed of carvacrol (61.08–83.37%), p-cymene (3.02–9.87%), and γ-terpinene (4.13–6.34%) [91] and *Myrtus communis EO* is largely dominated by monoterpene hydrocarbons, with α-pinene (24.3–59.0%) and 1,8-cineole (13.2–49.5%) [147]. This evidence easily demonstrates that the majority of compounds are terpenes, and they are responsible for the characteristic aromas and flavors of these plants. One of the notable properties of terpenes is their antibacterial activity. While the specific mechanisms of action may vary depending on the terpene and the bacterial species, some common mechanisms include disruption of cell membranes, inhibition of enzyme activity, and interference with bacterial growth and replication [148,149,150,151]. Recently, terpenes have been found to be effective inhibitors of efflux pumps in a diverse range of bacterial strains, suggesting their potential utility in drug development for addressing antibiotic resistance [149]. Using terpenes as a sole treatment for bacterial infections may not be sufficient; they may be more effective as part of a comprehensive approach that includes traditional antibiotics and other appropriate medical interventions.

The presence of monoterpenes (alpha-pinene, beta-pinene, limonene, and myrcene) and sesquiterpenes (beta-caryophyllene, caryophyllene oxide, and germacrene-D) is generally a significant common trait among these essential oils [91,145,146,147]. Another common trait in the chemical composition of these essential oils is the presence of oxygenated compounds. These compounds include alcohols, esters, ketones, and aldehydes. For instance, essential oils from *Salvia officinalis* and *Origanum vulgare* are rich in oxygenated monoterpenes, such as thujone and carvacrol, which contribute to their potent antimicrobial properties [152,153]. *Myrtus communis* oil contains myrtenol and 1,8-cineole [125,147,154], while *Cistus* species oils contain alpha-pinene and caryophyllene oxide as prominent oxygenated compounds [142].

Similarly, phenolic compounds are abundant in the essential oils of these plants and contribute to their antioxidant and antimicrobial activities. *Salvia officinalis* and *Origanum vulgare* oils are particularly rich in phenolic compounds, such as thymol and carvacrol, which have been extensively studied for their health benefits [155,156].

Terpenoids, a class of compounds derived from terpenes, are also present in these essential oils. Notable terpenoids found in the oils of these plants include terpinen-4-ol, linalool, and borneol. These compounds contribute to the overall aroma and therapeutic effects of the oils.

ChatGPT-aided analysis helped with the qualitative representation of the compounds mainly present in the most effective EOs, as shown in the following Table 3.

**Figure 4:**
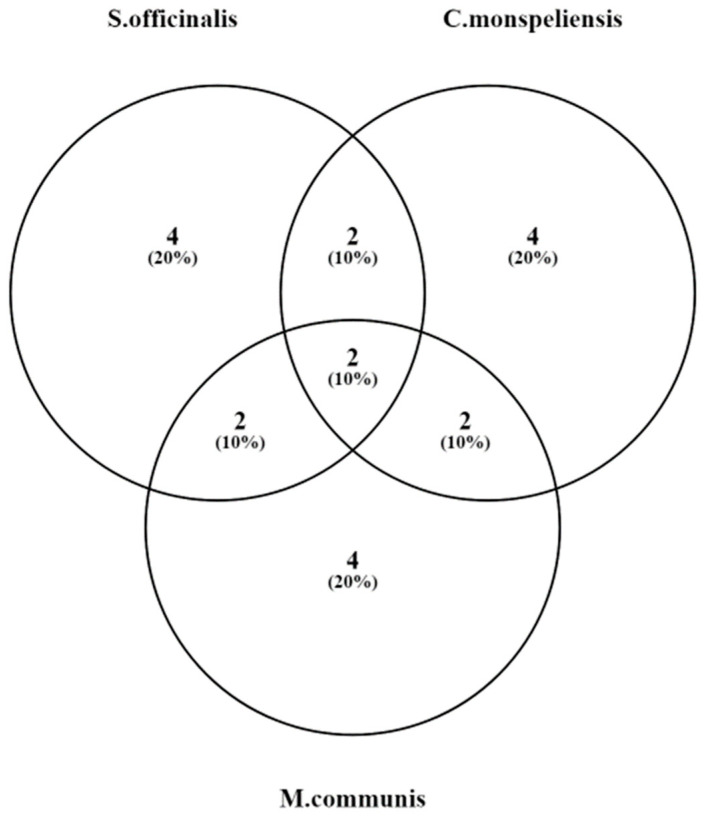
Venn diagram (ChatGPT-aided) representing the combinations of commonly shared compounds among the three most effective essential oils against *S. aureus*.

Limonene and α-Pinene are present in the three most effective EOs against *S. aureus* (Venn diagram in Figure 4). Limonene is a cyclic monoterpene with a pleasant citrus aroma known for its strong antimicrobial activity against various bacteria and fungi. Its mechanism of action may be attributed to its ability to disrupt the cell membranes of bacteria and fungi, leading to cell leakage and subsequent cell death. It also interferes with the enzymatic processes in microorganisms, affecting their growth and replication [148,151].

α-Pinene is a bicyclic monoterpene commonly found in the essential oils of pine trees and other coniferous plants. Like limonene, α-pinene has demonstrated significant antimicrobial activity related to its ability to disrupt bacterial membranes and interfere with their essential cellular processes [157,158]. Both compounds were demonstrated to be strongly active against *S. aureus* [148,151,157,158].

Phytocomplexes, defined as a blend of bioactive compounds, have the ability to work synergistically, targeting multiple receptors, aiding the molecules in reaching their intended destination, and slowing down the degradation of active compounds [159,160].

The superiority of plant phytocomplexes over individual molecules is evident from the decreased activity observed after fractionation [159,160]. Furthermore, it is widely acknowledged that there is a need for compounds that can synergistically enhance the effectiveness of existing antibiotics when combating drug-resistant bacteria [161,162,163].

Utilizing plant-based compound extracts could be beneficial in combating antibiotic resistance. Nevertheless, their eventual application requires cautious regulation and control to prevent the emergence of resistance mechanisms to less specific biocides such as antiseptics, disinfectants, and preservatives [164].

## 5. Conclusions

The published literature about plants with antimicrobial activity against *S. aureus* is extremely wide and heterogeneous, and for this reason, it is necessary to comprehensively analyze the published results to draw a conclusion. This work started from the idea of developing a Green Veterinary Pharmacology intervention to be used against the most relevant bacterial pathogens. However, before starting with an experimental (benchtop) approach, we considered it necessary to deeply understand what was already published to avoid repeating previously performed experiments and to avoid producing redundant experimental evidence.

The essential oils of *Salvia officinalis*, *Cistus monspeliensis*, *Cistus salviifolius*, *Origanum vulgare*, and *Myrtus communis* share common traits in their chemical composition, including the presence of monoterpenes, sesquiterpenes, oxygenated compounds, phenolic compounds, and terpenoids.

From obtained results herein described, it is possible to draw a conclusion about the most effective plants capable of inhibiting the growth of *S. aureus* that could be further tested for GVP interventions, such as the efficacy against isolates from animals.

## Figures and Tables

**Table 1:** List of plants growing in Italy producing extracts active against *S. aureus* [1].

Pathogen	Number of Active Plants	Plant Names and Reference
*Staphylococcus aureus*	39	*Cinnamomum* [14]; *Cinnamomum camphora* (L.) [15]; *Cistus monspeliensis* L. [16]; *Cistus salviifolius* L. [16]; *Cytinus hypocistis* (L.) L. [17]; *Limonium morisianum Arrigoni* [18]; *Myrtus communis* L. [19]; *Origanum vulgare* L. [20]; *Pistacia lentiscus* L. [21]; *Pistacia terebinthus* L. [22]; *Rosmarinus officinalis* L. [23]; *Salvia officinalis* L. [24]; *Thymus herba-barona Loise* L. [25]; *Thymus vulgaris* L. [26]; *Inula crithmoides* [27]; *Caralluma europaea* [28]; *Crocus sativus* [29]; *Helichrysum araxinum* [30]; *Schinus molle* (L.) [31]; *Cannabis sativa* [32]; *Centaurium erythraea* [33]; *Citrus medica* L., *Citrus bergamia*, and *Citrus medica* [34]; *Laurus nobilis* [35]; *Rubus ulmifolius* [36]; *Malus domestica* var. *Annurca* [37]; *Teucrium genus (Germander)* [38]; *Daucus carota* subsp. *maximus (Desf.)* [39]; *Cytinus* [40]; *T. vulgaris*, *Satureja montana* and *Coriandrum sativum* [41]; *Garlic* (*Allium sativum* L.) [42]; *Thymus vulgaris* L. [43]; *Rapa Catozza Napoletana (Brassica rapa* L. var. *rapa DC.)* [44]; *Calycotome villosa (Poiret)* [45]; *Juniperus* spp. [46]; *Hyssopus officinalis* [47]

**Table 2:** List of the number of records detected for each plant and filtered for inclusion and MICs annotation.

Plant	Total Records for Each Plant	Records Included
*Cinnamomum camphora*	23	8
*Cistus monspeliensis*	7	4
*Cistus salviifolius*	6	2
*Cytinus hypocistis*	3	1
*Myrtus communis*	69	14
*Origanum vulgare*	159	38
*Pistacia lentiscus*	32	4
*Pistacia terebinthus*	7	2
*Rosmarinus officinalis*	220	75
*Salvia officinalis*	102	9
*Laurus nobilis*	60	13
*Satureja montana*	15	8
*Coriandrum sativum*	11	7
*Garlic* (*Allium sativum* L.)	263	21
*Thymus vulgaris* L.	196	48
*Crocus sativus*	26	7
*Schinus molle*	28	8
*Cannabis sativa*	53	14
*Centaurium erythraea*	8	5
*Citrus medica* L.	12	5
*Citrus bergamia*	13	1

**Table 3:** Qualitative representation of the volatile compounds present in the EOs.

*Salvia officinalis*	*Cistus monspeliensis*	*Myrtus communis (Myrtle)*
1,8-Cineole	Camphene	1,8-Cineole
Borneol	Eugenol	Geraniol
Camphor	Geraniol	Limonene
Limonene	Limonene	Linalool
Linalool	Nerol	Methyl eugenol
α-Humulene	Sabinene	Myrtenol
α-Pinene	α-Pinene	Myrtenyl acetate
α-Thujone	α-Thujone	Nerol
β-Pinene	β-Pinene	α-Pinene
β-Thujone	δ-3-Carene	α-Terpineol

## Supplementary Materials

The following supporting information can be downloaded at: https://www.mdpi.com/article/10.3390/microorganisms11092264/s1, Table S1: Excel file with the references listed in the title.
